# Endoscopic T-tube placement in the management of lye-induced esophageal perforation: Case report of a safe treatment strategy

**DOI:** 10.1186/1754-9493-3-19

**Published:** 2009-08-14

**Authors:** Mary Aisling McMahon, Fardod O'Kelly, Kheng Tian Lim, Narayanasamy Ravi, John Vincent Reynolds

**Affiliations:** 1 Department of Clinical Surgery, Trinity Centre, Trinity College Dublin and St. James's Hospital, Dublin 8, Ireland

## Abstract

Esophageal perforation is associated with a significant risk of morbidity and mortality. We report herein a case of lye-induced esophageal perforation managed successfully by employing endoscopic T-tube placement with a successful outcome.

## Background

Esophageal perforation may represent a life-threatening emergency, and delay in diagnosis can result in a significant increase in morbidity and mortality. The diagnosis depends on a high degree of suspicion, recognition of clinical features, confirmation by contrast esophagography or endoscopy and appropriate treatment. The most common cause of esophageal perforation is iatrogenic injury. Other causes include spontaneous perforation (Boerhaave syndrome), caustic chemical ingestion, foreign body penetration, and trauma. The outcome after esophageal perforation is dependent on the cause and location of the injury, the presence of underlying esophageal disease, the interval between injury and initiation of treatment, and the patient's overall health. Mediastinal and intra-thoracic perforations identified within 24 hours are usually managed by surgical repair. Later presentations are managed depending on the condition of the patient, and approaches include chest tube drainage, exclusion and diversion, the insertion of endoscopic endoprosthesis, or thoracotomy with repair and debridement. Because of concern over the failure of primary repair, particularly where surgery is not performed within 24 hours, a controlled esophageal fistula may be established by the insertion of a T-tube at the site of perforation [1,2]. We adapted this principle and report herein the first case of endoscopic T-tube insertion which was an effective adjunct in the management of a patient who had a delayed diagnosis of esophageal perforation from caustic ingestion.

## Case presentation

**Figure 1:**
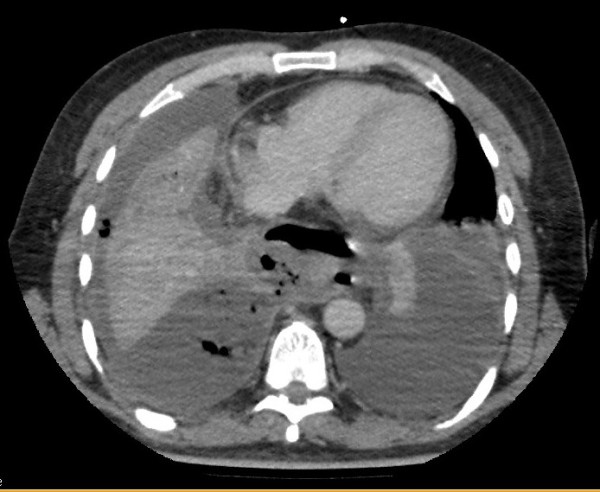
**CT Thorax showing bilateral pleural effusions and contrast leak in right hemithorax**.

A 51-year old gentleman with a background history of autistic spectrum disorder, depression and obsessive-compulsive disorder was admitted to the intensive care unit (ICU) following transfer from a referring hospital where he initially presented 6 days previously with a 24-hour history of vomiting and abdominal pain following ingestion of a lye detergent. There was no evidence of airway damage, and no initial evidence of esophageal perforation, and he was managed conservatively. On day 5 post admission he became septic, and imaging revealed air and contrast in the mediastinum and right thoracic cavity (Figure 1).

He was then transferred to this tertiary centre. On initial admission to ICU he was septic, with fever and hypotension, and signs of progressive respiratory failure. He was intubated and large-bore intercostal drains were inserted bilaterally. Esophagogastroduodenoscopy showed diffuse esophageal mucosal injury and but no obvious sign of perforation, and a repeat CT showed no obvious leak of contrast from the esophagus. A naso-jejunal tube was inserted for nutritional support. A percutaneous tracheostomy was inserted due to an anticipated requirement for prolonged ventilation. On day 30 in the ICU, gastric fluid leakage of 500 ml emerged from the right-sided chest drain. A repeat endoscopy revealed a large esophageal perforation in the lower esophagus opening into the right thorax, and the tip of the chest tube could be seen through this defect in the thoracic cavity.

**Figure 2:**
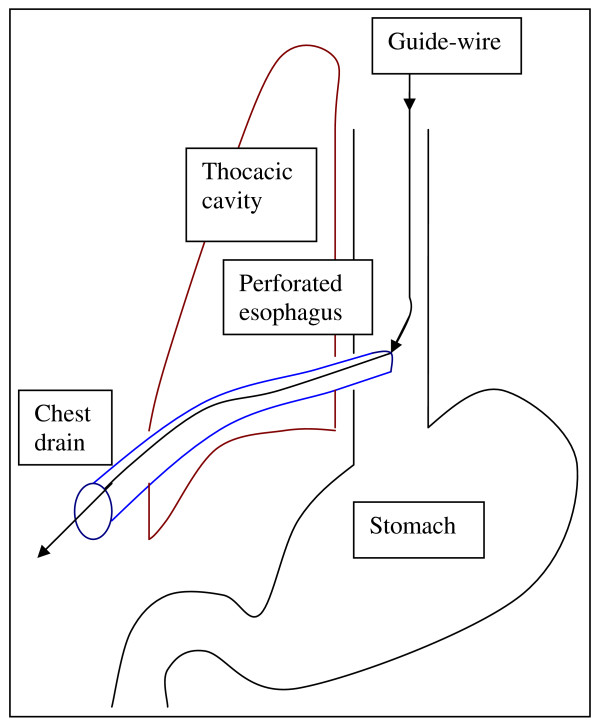
**Endoscopic antegrade insertion of guide-wire into the chest drain**.

**Figure 3:**
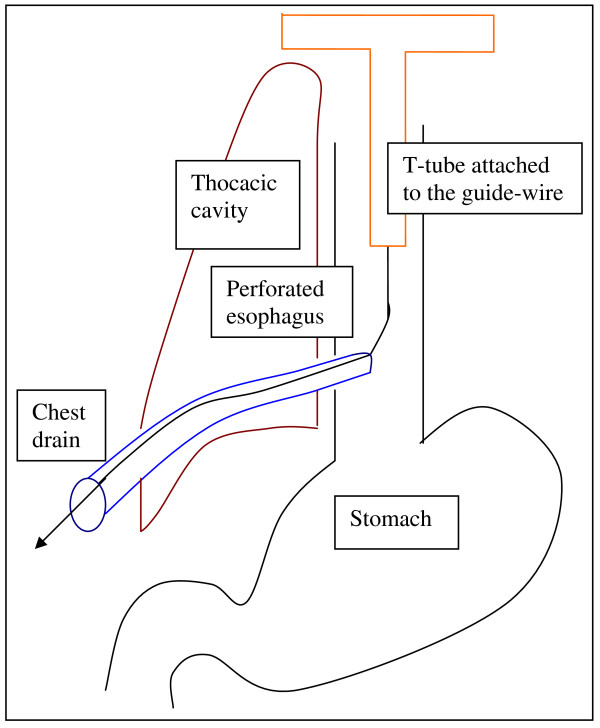
**Attachment of T-tube to the guide-wire which is then pulled through orally into the esophagus**.

**Figure 4:**
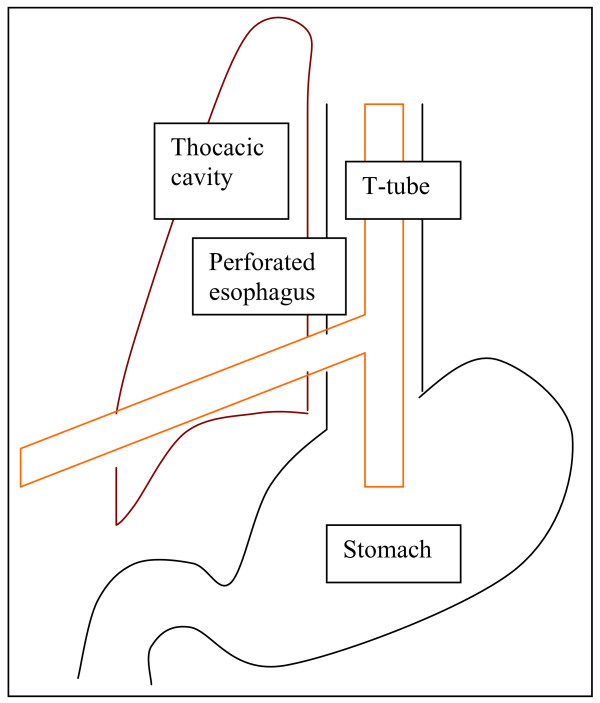
**Position of T-tube in the esophagus, stomach and through the esophageal perforation and thoracic cavity**.

**Figure 5:**
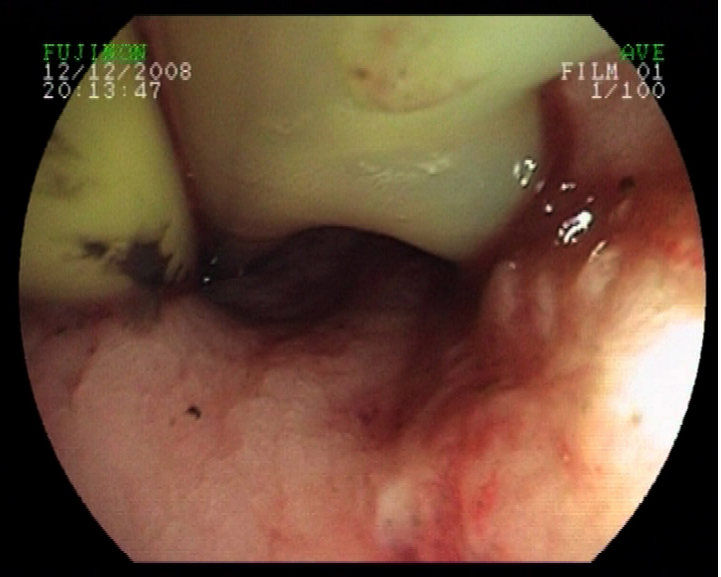
**Esophageal T-tube can be seen on the right and naso-jejunal feeding tube on the left**.

The decision was made to insert a T-tube endoscopically (see Additional file 1). The original intention was firstly, to insert the guide-wire in a retrograde direction through the chest drain into the thoracic cavity and secondly, to use the biopsy forceps that is passed endoscopically from the esophageal side to grasp the guide-wire and finally, to pull it out through the mouth. Since the tip of the chest drain was in continuity with the perforation, the guide-wire was firstly, inserted in an antegrade direction into the esophagus and out onto the chest wall through the right-sided chest drain (Figure 2). Secondly, the T-tube was attached to the guide-wire outside the mouth and then pulled through orally into the esophagus with the main stem of the T-tube pulled out onto the skin after the chest drain had been removed (Figure 3). Finally, the proximal and distal limbs of the T-tube were positioned in the esophagus and stomach respectively with the help of biopsy forceps (Figures 4 &5).

**Figure 6:**
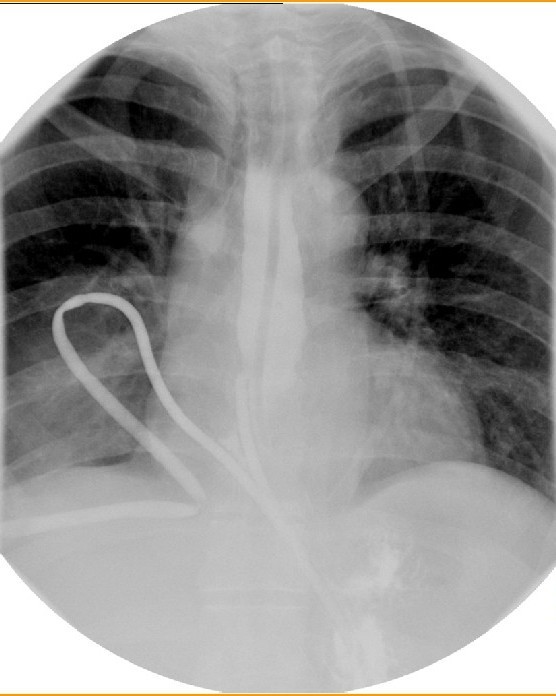
**T-tubogram demonstrated good passage of contrast in both limbs without any leak**.

A follow up tubogram showed good flow of contrast into the stomach and esophagus via the T limbs with no leak of contrast from the site of perforation (Figure 6). The T-tube was left on free drainage and allowed tract to mature. The patient's condition gradually recovered and he was transferred to the ward on day 45. Oral nutrition was tolerated well without sequelae. The T-tube was removed on day 60 and the patient discharged well. He remains well at 6 months of follow-up.

## Discussion

Esophageal perforation is associated with significant morbidity and mortality [3]. It is unassailable that the best outcomes will be achieved in specialist units with appropriate expertise in esophageal surgery, interventional radiology, and intensive care. The choice of management depends on a number of factors, including etiology, location of perforation, condition of esophageal tissue, and the overall health of the patient. The length of time from injury to diagnosis profoundly influences the management approach, with an early (<24 hrs) diagnosis often managed surgically in contrast to a more conservative approach for the majority of cases presenting later than 24 hrs.

Esophageal tissue is less amenable to repair after the first 24 hours. In a review, Brinster et al reported that primary repair of early esophageal perforations is associated with a low incidence of leak and a mortality rate of 10%, but that the mortality of surgery beyond 24 hours approaches 40%, and this may be associated with a high incidence of fistula [3]. A number of reports accordingly have encouraged the use of T-tube insertion for late presentations, with reports of its safety and efficacy, and some use it for all cases [2,4-7].

In this case, the delay in diagnosis and the initial assessment of a healed perforation suggested that a non-operative course was appropriate as long as the mediastinal and thoracic sepsis could be adequately drained. When gastric contents presented themselves via the chest drain a full month following the initial insult, we adapted this principle and were able simply to place the T-tube using the combination of access via endoscopy and the intercostal chest tube, thus establishing a controlled fistula. The exclusion criterion for this novel endoscopic treatment is acute esophageal perforation within first 24 hours. It is an option for delayed diagnosis of esophageal perforation. We acknowledge that the subsequent improvement in his clinical condition may have occurred in any case, and the perforation may eventually have healed, but we wish to highlight the ease of this technique and the benefits in terms of advancing his oral intake and a successful outcome.

## Conclusion

This case report highlights the safety, simplicity and efficacy of T-tube drainage in the management of delayed esophageal perforation, and this may represent a useful adjunct in the non-operative management of selected.

## Consent

Written informed consent was obtained from the patient for publication of this case report and any accompanying images. A copy of the written consent is available for review by the Editor-in-Chief of this journal.

## Competing interests

The authors declare that they have no competing interests.

## Authors' contributions

MAM and FO drafted the manuscript. KTL prepared the figures and movie clip, reviewed, amended and finalized the manuscript. NR provided technical information and reviewed the manuscript. JVR critically reviewed, amended and finalized the manuscript. All authors read and approved the final manuscript.

## Supplementary Material

Additional file 1 **Endoscopic esophageal T-tube insertion for perforated esophagus**. The movie clip shows the step by step techniques of endoscopic esophageal T-tube insertion for perforated esophagus. Click here for file
